# Molecular and Biological Aspects of Orthodontic Tooth Movement: Possibilities for Bioengineering Intervention: A Narrative Review

**DOI:** 10.3390/bioengineering10111275

**Published:** 2023-11-02

**Authors:** Ioannis A. Tsolakis, Isidora Christopoulou, Symeon Sitaras, Ioannis Lyros, Aliki Rontogianni, Maria Dalampira, Apostolos I. Tsolakis

**Affiliations:** 1 Department of Orthodontics, School of Dentistry, Faculty of Health Sciences, Aristotle University of Thessaloniki, 54124 Thessaloniki, Greece; 2 Department of Orthodontics, School of Dental Medicine, Case Western Reserve University, Cleveland, OH 44106, USA; apostso@otenet.gr; 3 Department of Orthodontics, School of Dentistry, National and Kapodistrian University of Athens, 11527 Athens, Greece; isidorachr@gmail.com (I.C.); yiannislyros@hotmail.com (I.L.); alikironto@yahoo.gr (A.R.); 4 Private Practice, 54124 Thessaloniki, Greece; sitsim93@gmail.com (S.S.); mdalabira@gmail.com (M.D.)

**Keywords:** orthodontic tooth movement, biomedical molecules, biomarkers, RANK, RANK-L OPG, vibration methods

## Abstract

Background: The current review’s goal is to examine, with a critical eye, the effect of various biomedical parameters on orthodontic tooth movement in an attempt to provide the reader with related mechanisms of this issue focusing on certain key points. Methods: This critical review was conducted using the following keywords in the search strategy: “biomedical molecules”, “biomarkers”, “orthodontics”, “orthodontic tooth movement”, “acceleration”, “gene therapy”, and “stem cells”. Cochrane Library, Medline (PubMed), and Scopus were the databases that were used for the electronic search. Studies published until June 2023 were considered. Results: The use of biomedical approaches in orthodontic tooth movement has been investigated via different procedures and applications. Surgical approaches, biomarkers affecting orthodontic tooth movement, different biological events and mechanisms, RANK, RANK-L, OPG molecular triad, and vibration methods are the basic parameters of biomedical interventions that are examined in the present review. Conclusions: The biomedical approach seems to offer a variety of applications to control orthodontic tooth movement. The scarcity of human studies, as well as the high cost and complexity of these methods, currently limit the available accurate data concerning this issue.

## 1. Introduction

Orthodontic tooth movement is a result of the resorption and apposition of bone through forces applied to the periodontal ligament (PDL) and alveolar bone. In recent years, there have been many studies regarding the effect of biomedical interventions on orthodontic tooth movement [1,2]. Surgical, pharmacological, and physical stimulation with conventional orthodontic force is reported to accelerate orthodontic tooth movement and shorten the treatment duration [3,4,5,6]. Orthodontic tooth movement can also be induced surgically using techniques including corticotomy, piezoelectric ultrasonic bone punctures, and alveolar decortication [3,4]. Moreover, mechanical stimulation such as low-intensity pulsed ultrasound (LIPUS) promotes fracture healing and enhances or even accelerates orthodontic tooth movement [6]. 

Orthodontic tooth movement is greatly influenced by osteoblasts, which are responsible for bone formation and apposition. Osteoblasts are osteogenic stem cells found in the skeleton that create the extracellular matrix that is high in type I collagen. Their activity is crucial for maintaining optimal remodeling and regeneration activity and achieving proper development and formation. The extracellular matrix is produced and organized by osteoblasts through the expression of a broad range of genes, which serves as their primary role. These genes produce regulatory, structural, and enzymatic proteins, including osteocalcin (OCN/BGLAP), osteonectin (ON/SPARC), osteopontin (OPN/SPP1), alkaline phosphatase (ALP/TNAP), bone sialoprotein (BSP/IBSP), and collagen type I (COL1A1, COL1A2). The secretion of osteoblasts is managed by an extended signaling pathway that is prompted by hormones, cytokines, prostaglandins, growth factors, and vitamins. The ability of osteoblasts to produce by themselves an extended range of molecules that can respond as autocrine, paracrine, and hormonal factors makes their role crucial in establishing the connection between the skeleton and the other organs. 

In addition, several biomarkers such as the molecular triad OPG/RANK/RANKL have been studied in regard to their capacity to affect orthodontic tooth movement [7,8]. However, to date, there are no universally accepted biomarkers for measuring and controlling orthodontic tooth movement. As the scarcity of the available studies does not permit the conduction of a systematic review, we decided to perform a narrative review examining multiple sides of the same subject. Hence, the purpose of the present review is to investigate, with a critical eye, the effect of various biological intervention parameters on orthodontic tooth movement in an attempt to provide the reader with a general idea of this issue focusing on certain key points.

## 2. Materials and Methods

With the exception of a few clinical research studies or reviews that exclusively focused on a specific area of biological approach in orthodontic tooth movement, there have not been many reviews examining various aspects of this problem at once until now. For these reasons, we chose to conduct a critical review utilizing a standard format to critically address a number of interesting topics.

This critical review was conducted using the following keywords in the search strategy: “biomedical molecules”, “biomarkers”, “orthodontics”, “orthodontic tooth movement”, “acceleration”, “gene therapy”, and “stem cells”. Cochrane Library, Medline (Pub-Med), and Scopus were the databases used for the electronic search. Table 1 shows the research strategy for PubMed. Studies published until June 2023 were searched for by two blinded researchers.

## 3. Results

**Figure 1:**
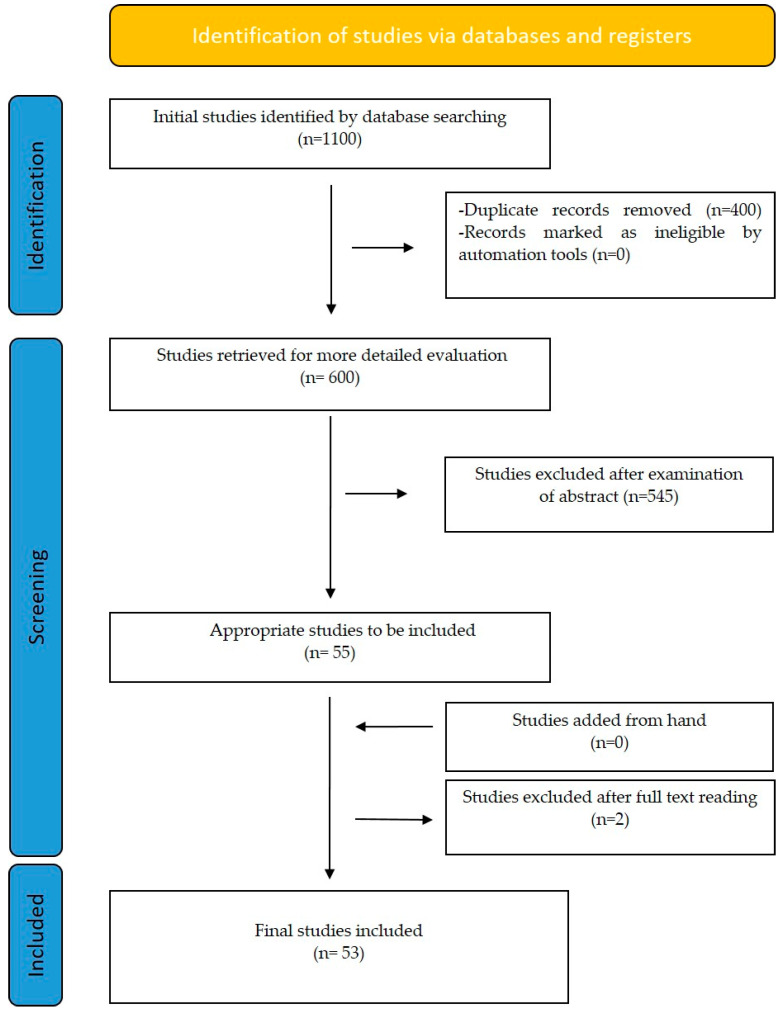
Flow diagram of the selection process for articles.

The use of a biomedical approach in orthodontic tooth movement has been investigated via different procedures and applications. Surgical approaches and biomarkers affecting orthodontic tooth movement are the basic parameters of biomedical intervention that are examined in the present review. Before thoroughly examining the effects of the biomedical approach on orthodontic tooth movement, it seems necessary to analyze the biological events responsible for orthodontic tooth movement as well as its mechanism. A flow diagram shows the selection process for the articles (Figure 1). The summary of our results is presented in Table 2 and Table 3.

### 3.1. Orthodontic Tooth Movement: Biological Events and Mechanisms

Orthodontic tooth movement is induced by mechanical stimuli and facilitated by remodeling of the periodontal ligament (PDL) and alveolar bone. The periodontal tissues that support the tooth root with its cementum-covered surface, such as the periodontal ligament (PDL), alveolar bone, and gingiva, are affected by orthodontic force as a mechanical stress. In addition to osteoclasts, osteoblasts, fibroblasts, mesenchymal stem cells (MSCs), and endothelial cells, the PDL is a multifunctional fibrous tissue that connects the cementum covering the tooth root and the alveolar bone. The PDL is the first, and extremely sensitive, receptor that senses the mechanical stimulations caused by the application of orthodontic force [9,10,11,12,13]. Following mechanical stimuli, orthodontic tooth movement starts taking place, incorporating three phases: (a) the lag period; (b) the tooth movement phase; and (c) the bone remodeling phase, which includes osteoclastic bone resorption and osteoblastic bone formation [14,15]. More specifically, when force is applied to a tooth, osteoclastic activity is encouraged on the pressure side of the tooth, and when PDL fibroblasts and MSCs have multiplied and differentiated, osteoblasts boost bone production on the tension side. In addition, mechanical force loading rapidly activates many cell signaling pathways in osteoblasts, including calcium (Ca^2+^), nitric oxide (NO), interleukin-1 (IL-1), and adenosine triphosphate (ATP). Orthodontic tooth movement results in the release of the mediators NO and IL. Fluid shear stress can activate the Ca^2+^ signaling system, which encourages ATP release, PGE2 secretion, and osteoblast development. Localized hypoxia and decreased blood flow in PDL happen concurrently on the compression side. Hypoxia-inducible factor 1 (HIF-1) is a transcription factor that activates VEGF and RANKL expression in PDL fibroblasts and osteoblasts, causing osteoclast differentiation and inducing bone resorption in the compression areas. The reduction in O_2_ tension stabilizes HIF-1. As a result, the tooth progresses in the desired direction, and the width and integrity of the PDL are preserved by a balance between bone apposition and resorption [14,15]. The remodeling of the alveolar bone surrounding the tooth’s root during orthodontic tooth movement makes it clear that bone resorption and creation are linked.

### 3.2. RANK, RANK-L, and OPG Molecular Triad

It has only recently been discovered that the receptor activator of nuclear factor-kappa (RANK), receptor activator of nuclear factor-kappa ligand (RANKL), and osteoprotegerin (OPG) systems play a crucial role in causing bone remodeling. This bone remodeling procedure involves the tumor necrosis factor (TNF)-related ligand (RANKL) and its two receptors (RANK and OPG) [16,17]. More specifically, RANKL controls the development and activation of osteoclasts, which explains how numerous hormones and cytokines have an osteo-resorptive effect. When RANKL binds to the RANK receptor on osteoclast lineage cells, it exerts its influence on the bone tissue. RANKL is expressed on the osteoblast cell lineage. This binding causes hematopoietic osteoclast precursors to quickly differentiate into adult osteoclasts. OPG, an osteoblast-produced receptor, faces up against RANK for RANKL binding [16,17]. The biological effects of OPG on bone cells include inhibition of osteoclastic differentiation, suppression of activation of matrix osteoclasts, and induction of apoptosis. It is evident that bone remodeling in orthodontic tooth movement is controlled by a balance between RANK–RANKL binding and OPG production [16,17,18]. 

### 3.3. Biomarkers

A prerequisite for the remodeling activities occurring in orthodontic tooth movement (OTM), and ultimately for tooth displacement, is the occurrence of an inflammatory process. Vascular and cellular changes were the first events to be described in the relevant literature, and a number of inflammatory mediators, growth factors, and neuropeptides have been demonstrated in the PDL. The early phase of tooth movement involves an acute inflammatory response characterized by periodontal vasodilation and migration of leukocytes out of the capillaries. Inflammatory mediators are released as chemical messengers following a mechanical stimulus, triggering the biologic processes associated with alveolar bone resorption and apposition [7,8]. It is true that patients with plaque accumulation following bracket placement and poor oral hygiene can induce an increase in inflammatory biomarker concentrations in oral fluids. In recent research, the PHP (patient hygiene performance index), PI (plaque index), and GI (gingival index) were significantly related to IL-6 concentrations in crevicular fluid, and it was found that an increase in the frequency of home oral hygiene decreased the concentration of IL-6 in the crevicular fluid. With regards to the same issue, it would be interesting to investigate the effect on these inflammatory biomarkers concerning the speed and efficacy of OTM [19]. Cytokines, which are released by mononuclear cells and leukocytes, are among the local biochemical mediators. Prostaglandins (PGs) and growth factors are just a couple of the substances that cytokines can stimulate the production and secretion of. By interacting directly or indirectly with bone cells, cytokines can affect bone formation, bone resorption, and orthodontic tooth movement [7,8,9]. The levels of inflammatory mediators (IL-1b, IL-6, TNF-a, and epidermal growth factor) seem to play an important role in the inflammatory phase, affecting the osteoclastic activity and the bone resorption rate. A study reveals that mechanical force induces a functional shift of the *Ccr2* macrophage cluster mediated by the NF-κB pathway, leading to pro-inflammatory activation and bone remodeling. This macrophage cluster may represent a potential target for the manipulation of OTM [20].

Another factor that seems to play an important role in OTM is the Runx2 gene, which has been examined in several studies reporting that mechanical stress activates Runx2 to promote osteogenesis in osteoblastic cells [21,22,23,24,25]. Experimental tooth movement seems to be delayed in Runx2^+/−^ mice, the animal model of CCD patients. These findings suggest that delayed tooth movement is associated with diminished bone formation on the tension side in Runx2^+/−^ mice. In contrast, Runx2 enhanced osteoclastic differentiation by activating the NF-κB ligand (RANKL) and OPG, which both are important factors for osteoclastogenesis [26]. As a result, Runx2 would definitely have some impact on bone resorption from the perspective of compression. However, more research is required to fully understand how osteoclasts work in Runx2^+/−^ mice. The relevant studies suggest that the association of Runx2 with mTORC2/Akt activation is of high importance for stretch-induced proliferation and differentiation to osteoblasts in osteogenesis during tooth movement.

### 3.4. Gene Therapy and Its Application in Alveolar Bone and OTM

Recent discoveries in the field of biology and genetics have not only permitted the control of hormones and the selective activation or deactivation of genes such as Runx2 but also the control of gene expression. In order to inject specific DNA/RNA fragments into the host or patient, the concept of gene therapy entails cloning them into a delivery system. Viral or non-viral vectors, such as liposomes, peptides, polymer particles, gene guns, and electric perforation, could be used as the delivery strategy. Gene therapy, as it is called, has a lot of dental applications [27]. Bones may be regenerated, remodeled, and repaired, unlike other dental hard tissues (such enamel and dentin) [28]. The processes of osteoinduction, osteoconduction, and osteoblast differentiation that result in the creation of the osteoid matrix can all be improved by gene therapy. Only the signaling molecules known as bone morphogenetic proteins (BMP-2, 4, and 7) can cause de novo bone production. The possibility of delivering the BMP-2 genes directly to the tissues via an adenoviral vector was demonstrated in a study focusing on this topic in an effort to promote bone growth and use BMPs in place of bone grafts [29]. This capacity of BMPs would be extremely useful in periodontal defects where OTM may be difficult or even harmful, depending on the depth and the geometry of the socket. In addition, another study found that after being infected with an adenoviral vector, a variety of cell types, including osteoblasts and non-osteogenic fibroblasts and myoblasts, can express the BMP-7 gene. When these cells are introduced into an osseous defect in vivo, they can then develop into bone-forming cells [30]. Although BMPs can cause osteo-induction individually, there is strong evidence that they can also work in collaboration to induce bone formation so that they can be successfully used in fracture healing. It has been shown that coordinated expression of BMPs 2, 3a, 4, 7, and 8 during fracture healing is important in both skeletal development and repair [31]. 

Regarding the effect of triad RANK, RANKL, and OPG to speed up and slow down orthodontic tooth movement in rats, two important investigations were conducted employing gene therapy using OPG and RANKL. The periodontal tissue received local RANKL gene transfer, which accelerated OTM by roughly 150% after 21 days without having any systemic side effects, thus decreasing treatment duration [31,32]. As a result, it was proposed that local RANKL gene transfer would be a potential method for shifting ankylosed teeth as well as for shortening orthodontic treatment. After 21 days of force application, local OPG gene transfer prevented tooth movement by roughly 50% in contrast to RANKL [31,32]. The effect of OPG gene transfer was local and did not affect bone mineral density of the tibia in the animals [31,32]. These capabilities offered by gene therapy may change the paradigm shift in orthodontic treatment, affecting both treatment time and orthodontic results [30,33]. Moreover, gene therapy has also shown promising results in diminishing the pain of OTM [34]. However, the available studies on this issue are still scarce.

### 3.5. Future of Genetic Manipulation of OTM

Gene targeting using endogenous microRNA (miRNA) has become a potent method for targeted gene delivery over the past ten years. The expression of genes is closely regulated by miRNAs, which are short, non-coding, and highly conserved RNA sequences that bind to their target region in the matching mRNAs. miRNA selection, delivery vehicle selection, system evaluation in cells, animal models, and clinical trials are all steps in the process of gene therapy using miRNAs [35]. Numerous miRNA expressions and functions in the PDL and the alveolus have been reported [36]. Several miRNAs respond to loading and force orientation in PDL and alveolar bone in various patterns of expression [37,38,39]. It has been demonstrated that miRNA-21 plays critical functions in PDL, osteoblasts, and osteoclasts. For the treatment of OTM, these microRNAs could be a promising gene therapy candidate.

The CRISPR/Cas9 system is another genome-editing technology that has lately attracted research interest. The CRISPR/Cas9 system is built on the CRISPR (clustered regularly interspaced short palindromic repeats) sequence and CRISPR associated (Cas) gene mechanism, which are essential for innate defense against bacteria and archaea and allow the organism to react to and eliminate invasive genetic material from their phages. The few and conflicting studies that are now accessible on this subject, however, offer little information, which needs to be confirmed by more studies in the same field in the future. 

The development of safer vectors or non-viral vector delivery systems, as well as the improvement of lentiviral vector-based techniques, are potential future areas for gene therapy. Before moving further with the application of human research, gene therapy techniques in the orthodontic field will need to show the safety and efficacy of the treatment concept in a number of fundamental cell culture and animal investigations. The next stage is to conduct clinical studies in order to test the effectiveness of the suggested treatments on both doctors and patients.

### 3.6. Invasive Methods: Procedures Affecting Orthodontic Tooth Movement

Numerous techniques to speed up orthodontic tooth movement (OTM) have been developed as a result of the advantages of reducing the length of orthodontic therapy. There is little evidence to support the effectiveness of invasive surgical procedures, including alveolar decortication, piezocision, corticision, and corticotomy, in hastening OTM [1,2,3,4]. There are currently few investigations on the biomechanical impact of corticotomy and osteotomy surgical bone cuts on orthodontic tooth movement [1,2,3,4]. The regional acceleratory phenomenon (RAP), which is the local and transient demineralization and remineralization happening in the alveolar bone during the wound healing period and first described by Frost, is thought to be connected to the biological consequences of corticotomy [26,40]. The common consensus seems to be that deeper surgical incisions cause more tooth movement. The amount of resistance experienced while moving teeth appears to be related to how much of the dentoalveolar structure has been surgically disrupted. Furthermore, the extent and the position of osteotomy or corticotomy can affect the mechanical responses of the dentoalveolar structures. The increased tooth movement might be explained by the RAP phenomenon. As far as the effects of low-intensity pulsed ultrasound is concerned, a single-dose application of LIPUS at 3 week intervals is ineffective in stimulating the OTM [41].

Corticotomy generates the RAP phenomenon, but osteotomy functions in a manner closer to distraction osteogenesis. Despite the fact that both corticotomy and osteotomy accelerate OTM, they do so through separate methods [42,43]. Periodontally accelerated osteogenic orthodontics, which is currently the most widely practiced clinical procedure to accelerate tooth movement, allows for faster tooth movement and involves alveolar corticotomy.

To avoid the drawbacks of surgical corticotomy, piezocision-assisted orthodontics was created. Due to the fact that many contemporary commercial piezotomes use high-frequency vibration, in addition to the direct bone damage induced by piezocision, vibration techniques may also promote bone remodeling.

### 3.7. Vibration Methods

Orthodontic tooth movement has been claimed to be accelerated by a variety of physical techniques, including low-level lasers, electromagnetic fields, photo-biomodulation, and vibration. To encourage tooth movement for orthodontic treatment, AcceleDent^TM^, a vibrating device, is currently being developed. There are publications demonstrating its efficiency in canine movement and leveling of the teeth during orthodontic treatment [43,44]. With no adverse effects, such as root resorption or pain, the static orthodontic force of 5.2 0.5 gf at 102.2 2.6 Hz for three minutes with supplemental vibration from AcceleDent^TM^ enhanced tooth movement in canine retraction [45].

### 3.8. Magnetic Fields

Magnetic fields, including static magnetic fields [39,40] and pulsed electromagnetic fields [46,47,48,49], have been shown to accelerate OTM in animal studies. According to histologic studies, alveolar bone remodeling is triggered by magnetic fields because bone cell activity is stimulated, and there is new bone deposition on the stress side [46,47,48,49]. What should be mentioned is that hyalinization in the PDL was reduced in a group treated with a static magnetic field, which also greatly contributed to the acceleration of tooth movement [47]. However, one study showed that the static magnetic field caused root resorption, raising doubts concerning the effectiveness and clinical applicability of this method [50]. In 2010, Showkatbakhsh et al. evaluated the effect of a pulsed electromagnetic field on orthodontic tooth movement. Their results suggested that the application of a pulsed electromagnetic field can accelerate orthodontic tooth movement [51]. Hence, further studies need to be conducted to determine the effect of magnetic fields on tooth movement and root resorption. 

### 3.9. Hormones

Hormones are known to influence OTM in several ways, either accelerating it or diminishing it. Specifically, parathyroid hormone (PTH) is the major hormone regulating bone remodeling and calcium homeostasis. Animal studies have shown that chronic local injections of PTH accelerate orthodontic tooth movement by about 1.6- to 2-fold and also significantly increase the number of osteoclasts [52]. However, it should be highlighted that these short-term studies did not determine the long-term effects of this hormone regarding OTM. 

Moreover, 1,25-dihydroxyvitamin D3 promotes calcium resorption in the small intestine. It also acts on bone cells by increasing bone remodeling [46]. Animal studies so far have indicated that a local injection of 1,25-dihydroxyvitamin D3 accelerates OTM by about 1.2- to 2.5-fold [53,54]. Histologic examination shows that 1,25-dihydroxyvitamin D3 stimulates the formation of osteoclasts in a dose-dependent manner, collaborating with the mechanical force and leading to significantly more alveolar bone resorption [48]. Like PTH, the safe use of this systemic factor in OTM should be further monitored.

PGs are small-scale, lipid-based, autocrine/paracrine inflammatory agents that control bone remodeling. Numerous investigations on animals have demonstrated that thromboxane A2, PGE1, or PGE2 analogs can speed up OTM when applied locally [55,56,57]. A local injection of PGE1 in human patients also proved to be effective in accelerating tooth movement by 1.6-fold [57]. Conversely, OTM seems to be impaired by nonsteroidal anti-inflammatory drugs, the compounds that inhibit the COX-1 and COX-2 enzymes that catalyze the rate-limiting step of PG formation [58]. It should be mentioned at this point that the major concern of using PGs in clinical practice is the pain reaction from patients since PGs are potent pain inducers. Another concern is the increased root resorption that is frequently associated with the reported accelerated tooth movement, as suggested by several studies [56,57,58]. 

### 3.10. Clinical Applications of Accelerating OTM 

Active orthodontic treatment often lasts 18 to 24 months, which is a significant time commitment. Since the 1890s, there has been a significant interest in accelerating tooth movement to reduce treatment time [59]. An average orthodontist saw 125 adult patients on average in 2014, compared to 41 adult patients in 1989, according to the 2015 AAO Survey. This is an enormous increase in recent years. Given that adults are not growing and that their local tissue metabolism and regeneration rates are substantially slower than those of adolescents, adult patients can gain the most from expedited orthodontic therapy. Adult patients are also more vulnerable to periodontal issues and other time-dependent adverse effects (such as issues with dental hygiene, root resorption, etc.). Accelerating treatment in adults has extra practical benefits as a result. Numerous surgical and nonsurgical methods have been used to hasten tooth movement because remodeling of the alveolar bone is a crucial part of orthodontic tooth movement. These methods work by interfering with the biological pathways that control the activity of bone cells (osteoclasts, osteoblasts, and osteocytes), which are the main components of remodeling.

In clinical practice, surgical methods to quicken orthodontic therapy have been used for more than a century. The initial methods involved creating a movable “bony block” by performing alveolar osteotomy alone or in combination with corticotomy. It was thought that by doing so, the resistance exerted by the surrounding cortical bone would be lessened, allowing the teeth to move more quickly. These procedures have been exceedingly intrusive and are linked to an increased risk of periodontal disease and tooth morbidity (mostly in situations where the interradicular space is smaller than 2 mm) [60,61]. Between the two techniques, selective alveolar corticotomy became the gold standard. Instead of movement of a bony block containing a tooth, Wilcko et al. were the first to hypothesize that rapid tooth movement following corticotomy may be caused by a demineralization–remineralization process that results in a regional acceleratory phenomenon (RAP) of bone remodeling [62]. In experimental research, mobility doubled during the RAP period at a pace of 1 mm every month [63].

Although surgical approaches have been found to accelerate orthodontic tooth movement, nonsurgical approaches have always been preferred by doctors and patients due to their decreased invasiveness. Prostaglandins, for example, are endogenously produced substances that affect bone remodeling. Exogenous application of these substances has specifically been tested for accelerating tooth movement, but the outcomes were disappointing because local administration of these agents was associated with an increased risk for root resorption and pain. Epidermal growth factor (EGF), parathyroid hormone (PTH), 1,25-dihydroxyvitamin D3, osteocalcin, and other new substances are now being tested on animals, and some have shown potential acceleration effects; however, further research is still needed to determine their safety and efficacy in humans. Due to its non-invasive nature and lack of discomfort, physical stimulation procedures are becoming more popular with both patients and orthodontists. Before they are widely used in clinical settings, their clinical efficacy must be proven, and more research from randomized studies is required.

## 4. Discussion

It is true that the target of each orthodontic treatment is the best possible outcome with the shortest possible treatment duration. Several studies have been conducted in an attempt to find methods to accelerate orthodontic tooth movement but with highly questionable results. As it has been previously mentioned, biomarkers that are proven to accelerate orthodontic tooth movement are still under investigation, and study results are controversial and inconclusive. RANKL, RUNX2, several cytokines (inflammatory and non-inflammatory), and specific cells seem to affect OTM by either accelerating or impairing it. Concerning RANKL, which has been investigated the most, previous studies of gene therapy in rats have shown that a constant, raised level of RANKL would allow for a continuous increase in OTM [64,65,66]. Unfortunately, gene therapy is currently inaccessible for orthodontic applications, but a localized and sustained dose of RANKL could potentially be a novel therapeutic approach to accelerate OTM and shorten treatment duration. However, it is apparent that these invasive studies are still difficult to conduct in human patients. 

As far as corticotomies and osteotomies are concerned, these surgical methods are known to affect OTM. However, due to the fact that they are highly invasive, causing trauma, pain, swelling, and discomfort in patients, human studies are scarce. Nevertheless, it should be highlighted that animal studies show promising results with few adverse effects. The same applies to low-laser stimulation or piezocision. For all these methods, it remains to be seen whether the results from human studies with a higher patient sample agree with the results from animal studies and how they can be adjusted to everyday orthodontic practice. 

Orthodontic tooth movement is a loading process that is carried out by various genes through genetic and epigenetic pathways governed by antagonistic and interplaying properties. It seems reasonable that periodontal tissue response to orthodontic loading is influenced by interpatient differences in the biologic properties of surrounding bone and connective tissues [32,67]. Following any changes in the local oral environment due to different loadings, as in the case of dental occlusion, normal pathophysiological processes trigger modeling and remodeling tissue pathways in normal and syndrome-free individuals.

The strength and nature of the applied forces are still a key determinant in orthodontic tooth movement in normal, healthy people. Frost’s “mechanostat” theory emphasizes the significance of strain levels and their impact on bone remodeling equilibrium [68]. Orthodontic tooth movement reactions to aging and abnormal general health conditions may be influenced by molecular mechanism intervention. Additionally, the type of mechanics used, as well as the strength and duration of the therapeutic forces used, may be influenced by altered secreted signaling molecules in blood and/or crevicular fluid.

The principle that is likely to change in the upcoming years will be based on a growing understanding of the biologic patterns of both bone formation and resorption, which will be achieved through interventions in the signaling pathways and molecules by genomic and hormonal regulation, and this will likely be followed by related breakthroughs. In patients who have hormonal and proteomic changes, one may alter the speed and quality of orthodontic tooth movement, leading to quicker and more stable results. In any instance, new bone and soft tissue remodeling paradigms may influence general body health and enable new treatment modalities, and as a result, new approaches may be applied in orthodontic treatment and craniofacial growth and development [69].

Following the rules of today’s bioengineering practice, it is necessary to determine the range and magnitude of applied stresses in alveolar and cortical bone as well to differentiate between orthodontic and orthopedic forces. There is currently a dearth of understanding regarding the relevance and size of the applied forces, which are secondary to all functional gene mechanisms that follow orthodontic tooth movement. Additionally, there is a knowledge gap regarding end tissue reactions, including distraction-like phenomena (DLP), Distal and remote alveolar bone responses (RAR), and regional acceleratory phenomena (RAP), , after orthodontic treatment in healthy individuals [70,71,72,73]. Therefore, it may be concluded that future pertinent studies must focus on local tissue bio-adaptability and reactivity as well as on the identification and classification of the various strain levels. In this way, a shorter course of treatment and better-quality and more stable orthodontic outcomes will be possible.

### Limitations

With regards to the limitations of the existing studies, it should be highlighted that characteristics, such as age, gender, bone metabolism, oral environment, and periodontal status, affect how orthodontic tooth movement occurs. Studies have shown that vibration can speed up the movement of orthodontic teeth in some cases but not in others. The issue with earlier studies may be that the tooth movement was not clearly assessed or studied, and the biological reason for tooth movement was not taken into consideration when choosing the vibration parameters and application method.

## 5. Conclusions

In conclusion, the biomedical approach seems to offer a variety of applications to control OTM. A localized and sustained dose of RANKL could potentially be a novel therapeutic approach to accelerate OTM and shorten treatment duration. Also, corticotomies, osteotomies, low-laser stimulation, and piezocision may accelerate orthodontic tooth movement (OTM) through interventions in bone remodeling. The scarcity of human studies as well as the high cost and complexity of these methods currently limit the available accurate data concerning this issue. More high-quality studies are needed to enrich our current knowledge and to offer the possibility for an evidence-based application of these methods in the near future.

## Figures and Tables

**Table 1:** The search strategy for PubMed.

“Orthodontics” [Major] and biomarkers	395 results
Orthodontic tooth movement [MeSH Major Topic] AND acceleration	375 results
Orthodontic tooth movement [MeSH Major Topic] AND gene therapy	139 results
Orthodontic tooth movement [MeSH Major Topic] AND stem cells	78 results
Orthodontic tooth movement [MeSH Major Topic] AND biomarkers	113 results

**Table 2:** Summary of results concerning methods affecting OTM.

Methods Affecting OTM	Accelerate OTM	Conflicting Results
RANKL		**+**
OPG		**+**
Inflammatory mediators (IL-1b, IL-6, TNF-a, epidermal growth factor)	**+**	
Runx2 gene	**+**	
Corticotomy	**+**	
Osteotomy	**+**	
Piezocision		**+**
AcceleDent^TM^	**+**	
Low-laser stimulation		**+**
PTH	**+**	
Prostaglandins (PGs)	**+**	
1,25-dihydroxyvitamin D3	**+**	
Magnetic fields		**+**

**Table 3:** Studies investigating different methods accelerating OTM.

Methods Affecting OTM	Animal Studies	Human Studies	Both Kinds of Studies
RANKL	**+**		
OPG	**+**		
Inflammatory mediators (IL-1b, IL-6, TNF-a, epidermal growth factor)	**+**		
Runx2 gene	**+**		
Corticotomy			**+**
Osteotomy			**+**
Piezocision			**+**
AcceleDent^TM^		**+**	
Low-laser stimulation			**+**
PTH	**+**		
Prostaglandins (PGs)	**+**		
1,25-dihydroxyvitamin D3	**+**		
Magnetic fields	**+**		
